# Glass Transition Temperature of PLGA Particles and the Influence on Drug Delivery Applications

**DOI:** 10.3390/polym14050993

**Published:** 2022-02-28

**Authors:** Guangliang Liu, Kathleen McEnnis

**Affiliations:** Otto H. York Department of Chemical and Materials Engineering, New Jersey Institute of Technology, Newark, NJ 07102, USA; gl242@njit.edu

**Keywords:** glass transition temperature, PLGA copolymers, drug delivery, nanoparticles

## Abstract

Over recent decades, poly(lactic-co-glycolic acid) (PLGA) based nano- and micro- drug delivery vehicles have been rapidly developed since PLGA was approved by the Food and Drug Administration (FDA). Common factors that influence PLGA particle properties have been extensively studied by researchers, such as particle size, polydispersity index (PDI), surface morphology, zeta potential, and drug loading efficiency. These properties have all been found to be key factors for determining the drug release kinetics of the drug delivery particles. For drug delivery applications the drug release behavior is a critical property, and PLGA drug delivery systems are still plagued with the issue of burst release when a large portion of the drug is suddenly released from the particle rather than the controlled release the particles are designed for. Other properties of the particles can play a role in the drug release behavior, such as the glass transition temperature (*T_g_*). The *T_g_*, however, is an underreported property of current PLGA based drug delivery systems. This review summarizes the basic knowledge of the glass transition temperature in PLGA particles, the factors that influence the *T_g_*, the effect of *T_g_* on drug release behavior, and presents the recent awareness of the influence of *T_g_* on drug delivery applications.

## 1. Introduction

**Figure 1:**
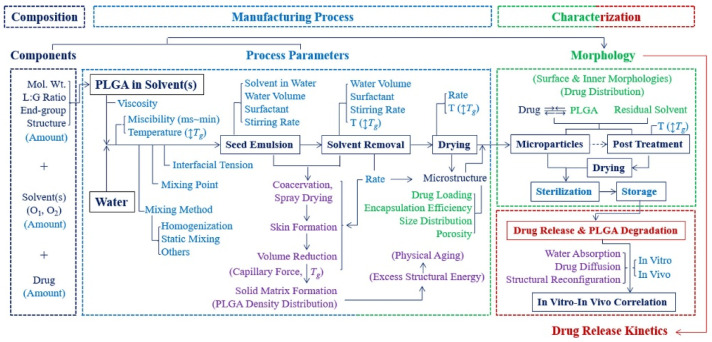
Flow chart of steps of manufacturing PLGA particles and their influence on particle properties [37]. Reprinted from Journal of Controlled Release, 329, Park, K.; Otte, A.; Sharifi, F.; Garner, J.; Skidmore, S.; Park, H.; Jhon, Y.K.; Qin, B.; Wang, Y., Formulation composition, manufacturing process, and characterization of poly(lactide-co-glycolide) microparticles, 1150–1161, Copyright (2021), with permission from Elsevier.

The application of polymeric particles in drug delivery has been rapidly developed in the past several decades [1,2,3,4,5,6,7,8,9]. Particle-based therapeutics offer considerable benefits compared with traditional pharmaceuticals, such as controlling release rates, overcoming biological barriers, delivering hydrophobic drugs, and targeting specific sites [10,11,12,13,14,15,16,17,18]. Polymeric particles guard the encapsulated drugs from enzymatic reactions in order to prolong the half-life of the encapsulated drugs [19,20,21]. The tunable size of the polymeric particles enables the travel through cell membrane barriers [22,23,24]. Diverse manufacturing approaches and surface modifications offer opportunities for the polymeric particles to reach the desired organ, tissue, and cells, thus minimizing the toxicity at other sites [25,26,27]. All these benefits make polymeric particles a promising drug delivery strategy. Poly(lactic-co-glycolic acid) (PLGA) has been proven to be a successful polymeric drug carrier and widely used in drug delivery, tissue engineering, and cancer therapies [28,29] due to its biocompatibility and biodegradability. Currently, more than 20 different PLGA formulations have been approved by the U.S. FDA [30]. PLGA undergoes a hydrolysis process in body fluid and generates biodegradable metabolite substances, lactic acid and glycolic acid, which can be eliminated by the human body [31]. Additionally, the availability of different PLGA polymer degradation rates, ranging from days to months, can be used to design an appropriate release profile, keeping the drug concentration between maximum toxic concentration (MTC) and minimum effective concentration (MEC), and increasing patient compliance. In order to achieve promised pharmacodynamics, biodistribution, and toxicity levels, the key physicochemical properties need to be appropriately studied. Particle size, size distribution, surface morphology, zeta potential, and loading efficiency are most commonly characterized because these parameters are the key factors that determine the drug release behaviors [32,33]. It has been proposed, however, that the glass transition temperature (*T_g_*) will also impact the drug release behavior of polymeric nanoparticles [34,35,36]. During this transition, the increased polymer chain mobility allows the drug molecule to escape from the polymer chain entanglement, resulting in an increased release rate. Notably, during the preparation of PLGA particles, the nature of PLGA copolymer, reactant components, and manufacturing process will contribute to changes in the *T_g_* (Figure 1) [37].

Glass transition temperature (*T_g_*) is usually defined as the temperature range where the polymer transitions from a hard glassy state to a relative rubbery state and is normally detected by the rapid change in heat capacity, specific volume, or stiffness. The *T_g_* is an important indicator of the physical properties of semi-crystalline and amorphous polymers. During the glass transition, the disordered chains in the amorphous portion start to escape the entanglements, increasing the polymer mobility on a macro scale which results in a soft rubbery substance. It has been well studied that during the glass transition, specific enthalpy, specific volume, thermal expansivity, motions of the polymer chain, and other parameters experience a dramatic change [38,39,40,41,42]. Notably, previous literature demonstrated that the *T_g_* of drug loaded PLGA particles ranges from 30 °C to 60 °C [43,44], indicating that PLGA particles could undergo a glass transition in a 37 °C drug release environment. The substantial change in PLGA particle physicochemical properties could lead to a different drug release rate from the particle matrix and an uncontrolled release profile. Nevertheless, even though the drug release kinetics has been frequently investigated by researchers with respect to the physicochemical properties of PLGA copolymer, drug type, manufacturing process, and post-treatment, there is significantly less literature focusing on associating drug release kinetics with the *T_g_* of PLGA particles.

**Figure 2:**
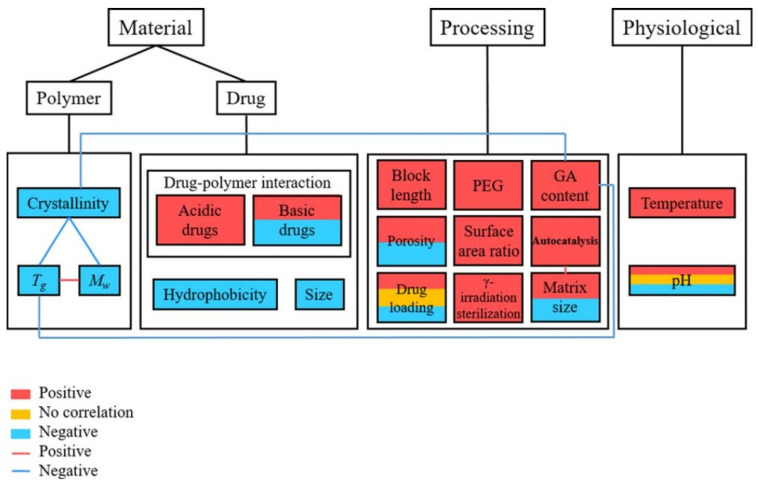
Link between the related parameters and the rate of drug release from PLGA carriers [46]. Reproduced with permission from Xu, Y. et al. Journal of Biomedical Materials Research Part B: Applied Biomaterials, published by John Wiley and Sons, Copyright 2017.

Quite a few reviews have recently been reported about PLGA nanoparticles in drug delivery. Mir et al. summarized the application of PLGA nano carriers in cardiovascular diseases, inflammatory disease, neurodegenerative diseases as well as cancer therapy and theragnostic [45]. Xu et al. provides a review on experimental observations and theoretical models, inferring the relation between the manufacturing factors and drug release profiles (Figure 2). These include the inherent properties of the PLGA polymer, influence of the drug loaded into the particle, processing parameters, and release environment [46]. Ding et al. presented the approaches of PLGA particle preparation, which could be classified as emulsification-solvent evaporation, nanoprecipitation, microfluidics, spray-drying, and phase separation [47]. Rezvantalab et al. summarized passive targeting, active targeting, and magnetic targeting for PLGA drug delivery nanoparticles for cancer treatment. In addition, PLGA nanoparticles can be used with other therapies, such as magnetic hyperthermia, photodynamic and photothermal therapy, and gene therapy [48]. Ghitman et al. provided the comparison between the traditional approach of preparing PLGA-lipid nano vehicles and novel approaches, which were soft lithography and spray drying. Ghitman’s review highlights the current challenges to fully understand the physicochemical properties of the nanocarriers and the interaction of targeting sites to determine the toxicity level and clinic safety [49]. Cunha et al. investigated the application of PLGA nanocarriers in neurodegenerative diseases, specifically the potential for PLGA nanocarriers to transport neuroprotective medicines across the blood-brain barrier [50]. Though many reviews exist exploring PLGA’s role as a drug delivery vehicle, none exist that take the glass transition temperature of the particles into account. This review summaries the factors that influence the *T_g_* of the PLGA copolymer, bare particles, and drug loaded particles. In addition, the connection of glass transition of PLGA particles and drug release behavior are discussed in terms of the mobility of PLGA particles, the physical ageing effect, and surface reconfiguration.

## 2. Glass Transition Temperature of PLGA Particles

### 2.1. PLGA Copolymer

**Figure 3:**
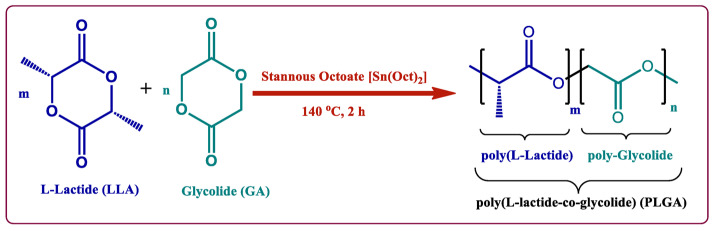
Copolymerization of PLGA by ring-opening method [52]. Reproduced from Butreddy, A. et al., International Journal of Molecular Sciences, 22, 2021, under Creative Commons Attribution 4.0 International License (http://creativecommons.org/licenses/by/4.0/ (accessed on 24 February 2022)).

PLGA is a linear random copolymer consisting of D,L-lactide and glycolide, which is usually prepared by polycondensation reaction and ring-opening polymerization of the two monomers (Figure 3) [51,52]. The physicochemical properties of the PLGA copolymer used in preparation are the determining factors of the properties of the PLGA particles, including monomer ratio, molecular weight, crystallinity, and end groups [35,53]. In addition, the final PLGA products are also affected by the approaches, reaction environment, and process parameters [54]. The molar ratio of the two monomers in the PLGA chains determines many physicochemical properties, such as the glass transition temperature, degradation rate, hydrophobicity, and degree of crystallinity [55,56]. In general, the *T_g_* of PLGA increases when the copolymer has a rich content of PLA. Peter In Pyo et al. reported that, among four different ratio PLGAs, PLGA with a ratio of lactide to glycolide of 90:10 (PLGA90:10) had the highest *T_g_* while PLGA with a ratio of 50:50 (PLGA50:50) had the lowest *T_g_* of 35.7 °C [57]. Brostow et al. developed an equation to predict the glass transition temperature of physical mixtures of binary systems and copolymers [58],

(1) $$T_{g}=x_{1}T_{g1}+\left(1-x_{1}\right)T_{g2}+x_{1}\left(1-x_{1}\right)\times \left[a_{0}+a_{1}\left(2x_{1}-1\right)+a_{2}{\left(2x_{1}-1\right)}^{2}+a_{3}{\left(2x_{1}-1\right)}^{3}\right]$$

where $T_{g}$ is the glass transition temperature of the given sample, $x_{1}$ is the weight fraction of component 1, $T_{g1}$ is the glass transition temperature of component 1, $x_{2}$ is the weight fraction of component 1, $T_{g2}$ is the glass transition temperature of component 2, $a_{0}$, $a_{1}$, $a_{2}$, and $a_{3}$ are parameters for the given copolymer or binary system.

It has also been reported that PLGA50:50 has the fastest degradation rate, which is due to the high percentage of hydrophilic glycolide, enabling water to penetrate the particle matrix and promote hydrolysis [59,60]. The Flory-Fox equation is a well-known empirical equation that describes the relationship between the number-average molecular weight and glass transition temperature, which was reported by Thomas et al. in 1950 [61]:

(2) $$T_{g}=T_{g,\infty }-\frac{K}{M_{n}}$$

where $T_{g,\infty }$ is the highest glass transition temperature for a given polymer under the theoretical condition that the molecular weight is infinitely high, *K* is an empirical parameter for a given polymer sample which is related to the free volume, and $M_{n}$ is the number-average molecular weight.

Briefly, *T_g_* has a positive correlation with polymer molecular weight. As the polymer chains become longer, the concentration of chain ends decreases in a unit volume resulting in less free volume between chain ends, thus the *T_g_* becomes higher [62]. Lee et al. illustrated that PLGA with molecular weight (MW) of 8000 g/mol has a *T_g_* of 42.17 °C, and as the MW increased to 110,000 g/mol, the *T_g_* rose to 52.62 °C [63]. Additionally, the crystallinity and the mobility of the polymer chain ends have a significant impact on free volume, and the *T_g_* rises as the degree of crystallinity grows or as the density of end groups decreases [64,65,66].

### 2.2. Glass Transition Temperature of Polymeric Particles

**Figure 4:**
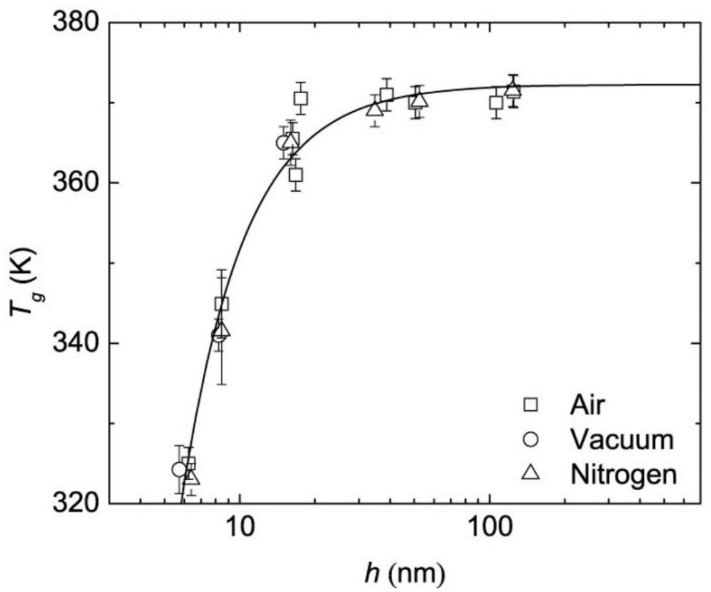
*T_g_* of PS thin films under different measuring environment [68]. Reprinted by permission from Springer Nature Customer Service Centre GmbH: Springer, The European Physical Journal E, Effect of atmosphere on reductions in the glass transition of thin polystyrene films, Raegen, A.N.; Massa, M.V.; Forrest, J.A.; Dalnoki-Veress, K., 2008.

When considering properties of substances at a nanoscale level, it is expected that the properties will be different from those of the bulk material, often because of the greater surface-to-volume ratio the nano substances have. Keddie’s group reported the first systematic study on the size-dependent glass transition temperature of thin polystyrene films supported by silicon substrates. In their work, three different molecular weight polystyrenes were used to create thin films and their *T_g_* was measured by ellipsometry. It was found that the *T_g_* dropped substantially when the film became thinner [67]. Raegen et al. further investigated PS thin films on substrates under ambient, dry nitrogen, and vacuum environments (Figure 4) [68]. For all experiments, the *T_g_* drop appeared with decreasing film thickness suggesting that the *T_g_* reduction in PS thin films was an intrinsic property. The surface area to volume was greatly reduced in these thin films, however, the two surfaces were different: one was the supporting substrate, and the other was a free surface. In order to reduce the inequality from the free surface and interface of the thin film and to better understand the interfacial effect on a polymer’s *T_g_*, spherical nanoparticles have been investigated by several research groups because a 3-dimensional geometry reduces the interface to one and the increase of the surface area to volume ratio will exhibit more obvious interfacial effects. Zhang et al. prepared polystyrene nanoparticles (PS NPs) of different sizes and the *T_g_* of the PS NPs suspended in water was measured by MDSC [69]. The results agreed with the trend of PS thin films, in which the PS nanoparticles of extremely small size will show a significant reduction in *T_g_*. It was well accepted that the *T_g_* reduction was caused by an enhanced mobile layer on the surface [67]. To further prove the interfacial effects on *T_g_* shift, they synthesized PS/silica core-shell structural nanoparticles, for which the silica on the surface was defined as a hard shell. It was observed that the silica capped nanoparticle samples did not have a size dependent *T_g_* reduction, which reinforced the conclusion that the mobile layer formed on the free surface will cause the *T_g_* shift.

**Figure 5:**
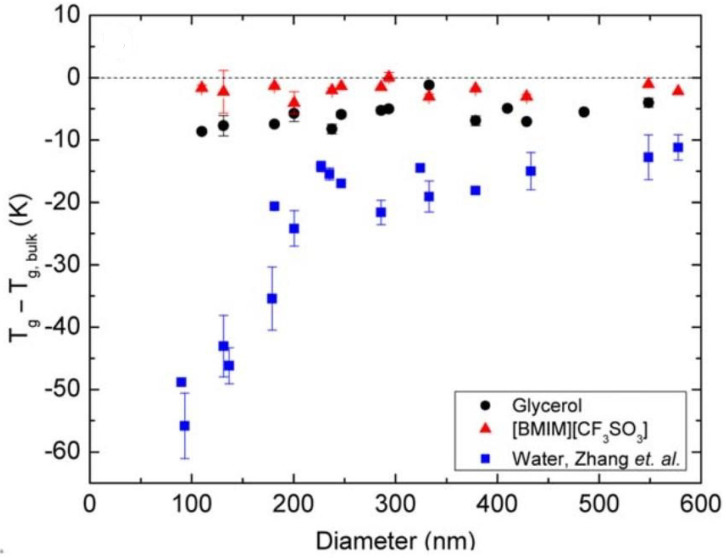
PS nanoparticles suspended in various liquids [70]. Reproduced with permission from Christie, D. et al. Journal of Polymer Science Part B: Polymer Physics, published by John Wiley and Sons, Copyright 2016.

Christie et al. investigated the effects of the measurement environment on *T_g_* by measuring the *T_g_* of PS nanoparticles suspended in three different liquids: glycerol, ionic liquid (1-butyl-3-methylimidazolium trifluoromethanesulfonate, [BMIM][CF_3_SO_3_]), and water [70]. As shown in Figure 5, the *T_g_* reduction of PS nanoparticles suspended in water, ionic liquid and glycerol will have a strong, independent, and weak correlation with the size. Also, the *T_g_* reduction from particles suspended in water will be similar to those measured in air, because the interfaces of water-PS and air-PS are considered “soft” due to their low viscosities compared with the polymer. The higher viscosity of glycerol, however, will inhibit the mobility of the glycerol-PS interface, resulting in a relatively inert polymer chain in the mobile layer. When considering the suspension in ionic liquid, ionic interactions dominate the mobility in the mobile layer because the positively charged [BMIM] molecule will anchor onto the negatively charged PS surface, inhibiting the mobility of the polymer chains at the interface.

Feng et al. proposed investigation in aqueous environments by preparing PS nanoparticles from a nonionic surfactant (Brij 98) and an anionic surfactant (sodium dodecyl benzene sulfonate (SDBS)) as well as surfactant-free particles [71]. A substantial reduction in *T_g_* with decreasing size of surfactant-free particles was observed which corresponded to previous studies. Nanoparticle surface softness is critical in *T_g_* characterization because of the high surface area to volume ratio in nano-size materials.

These studies demonstrate that polymeric particles under confinement exhibit variations in *T_g_* as a result of surface and interfacial effects. Due to the challenges associated with residual surfactant, size distributions, and other factors, the size-*T_g_* correlation has not been conducted explicitly on PLGA particles; nonetheless, similar trends are expected to occur in PLGA particles.

### 2.3. Drug Effect

**Figure 6:**
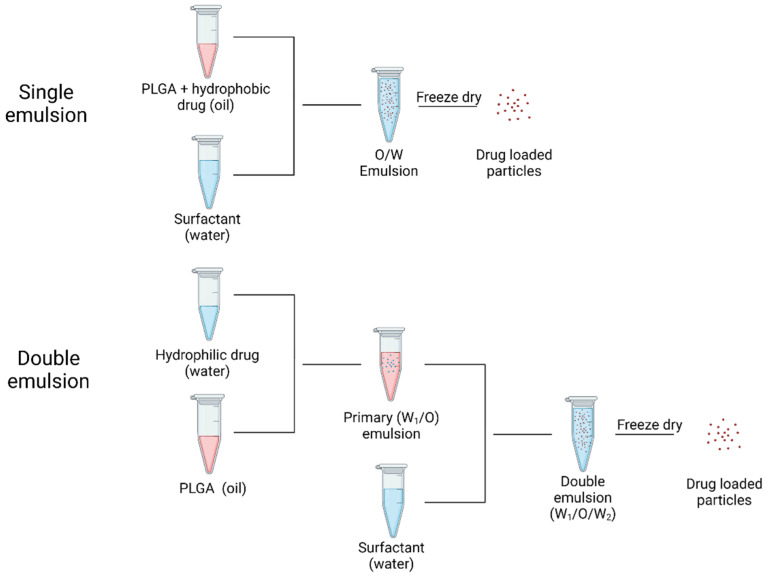
Processes of loading hydrophobic drug (single emulsion) and hydrophilic drug (double emulsion). Created in Biorender.com (accessed on 24 February 2022).

PLGA particles are extensively employed for a broad range of drugs, including hydrophobic and hydrophilic drugs. Drugs that are hydrophobic are easier to encapsulate in PLGA than those that are hydrophilic. Hydrophilic medicines often have lower drug loading efficiencies because the drug molecules enter the aqueous phase before the PLGA chains form into particles [72]. For loading hydrophobic and hydrophilic drugs into PLGA microparticles, the most extensively utilized methods are emulsion-evaporation technique (oil/water or water/oil/water) (Figure 6) [73,74,75]. The single emulsion technique involves an organic phase which contains PLGA polymer and the hydrophobic drug in a suitable organic solvent and an aqueous phase which contains a stabilizer. Mechanical force provided by ultrasonication is utilized to form an oil in water emulsion, and the organic solvent is then extracted to solidify PLGA particles [76]. On the other hand, hydrophilic drugs are usually encapsulated by a double emulsion technique to prevent diffusion of the drug into the aqueous phase. The inner water phase containing the hydrophilic drug is added into the PLGA solution to form a primary water-in-oil emulsion. Then the primary emulsion is injected into the outer water phase with the presence of a stabilizer to create a double emulsion. The final step is similar to the single emulsion process, which is to evaporate the organic solvent to obtain PLGA particles [77]. Another conventional preparation of PLGA particles is nanoprecipitation, which involves mixing a miscible solvent and stabilizer in water [77]. During the diffusion of organic solvent into the aqueous phase, nucleation, nuclei growth, and aggregation are expected to occur in order to form final particles [78]. Microfluidic technology is a novel method to produce narrow size distribution PLGA particles with high drug encapsulation. A microfluidic chip is made up of micro size channels which ensures the mixing of inlet flows to be completed within milliseconds [33]. Electrospray jetting can also be utilized to prepare PLGA particles. Electrospray jetting usually consists of a high voltage source, syringe pump, and collector. By adjusting the voltage, distance between collector and syringe, and flow rate, a Taylor-cone forms at the needle, which results in a stable spray. Solvent in the small droplets experience evaporation and solid particles reach the collector [79].

**Figure 7:**
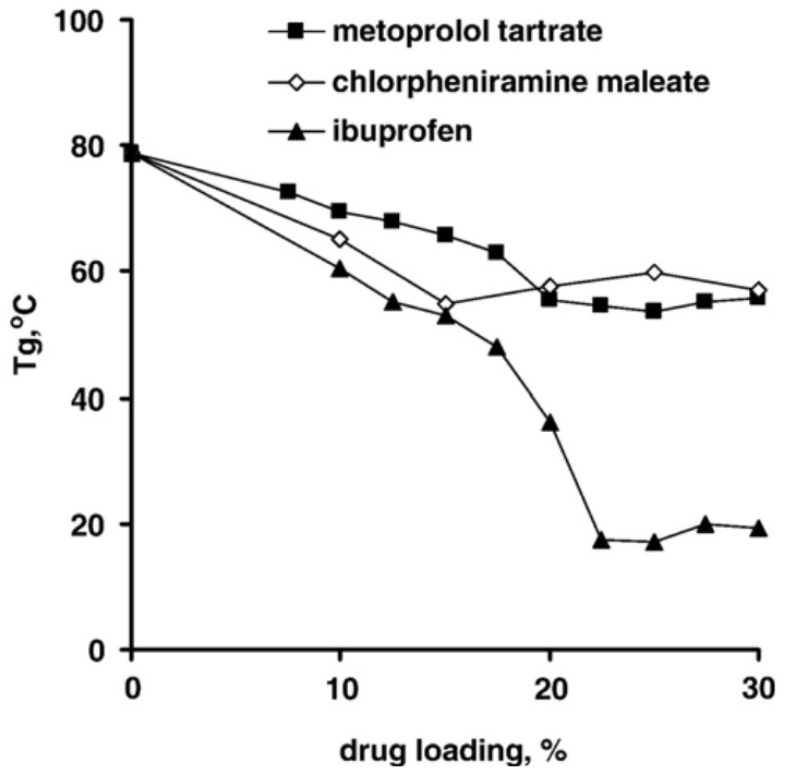
Plasticizing effects of drug types and drug loading efficiency [84]. Reprinted from Journal of Controlled Release, 115, Siepmann, F.; Le Brun, V.; Siepmann, J., Drugs acting as plasticizers in polymeric systems: A quantitative treatment, 298–306, Copyright (2006), with permission from Elsevier.

The drug type is tightly associated with drug release behavior, as hydrophobic molecules have a significantly lower degree of initial burst release than other pharmaceuticals due to their poor water solubility [80,81,82]. Steven et al. demonstrated the influence of a hydrophilic drug (aspirin) and a hydrophobic drug (haloperidol) on PLGA matrices release behavior, and the results showed that the drug with higher water solubility (aspirin) would give a relatively higher diffusion efficiency [83]. Apart from the influence of drug release behavior, the drug type will also determine the glass transition temperature of PLGA particles. Svenja et al. encapsulated flurbiprofen into 200 nm PLGA nanoparticles, and found that as the encapsulation efficiency increased, the *T_g_* of PLGA nanoparticles decreased from 28.8 °C to 19.9 °C, and that the overall mobility was improved by a higher flurbiprofen loading efficiency. Furthermore, they prepared mTHPP-loaded PLGA nanoparticles and measured the *T_g_*, which turned out to have no effect compared with the unloaded PLGA particles. Due to the higher molecular weight of mTHPP molecules, the rigid chemical structure, and relatively hydrophobic compounds, the mTHPP is unable to form a tight association with the polymer, preventing the polymer chain from becoming more mobile [34]. In order to investigate the plasticizing effect of different drugs in polymeric system, Siepmann et al. prepared thin films with metoprolol tartrate, chlorpheniramine maleate, and ibuprofen [84]. The experimental data illustrated that *T_g_* of the thin films decreased with the increased drug loading efficiency, which demonstrated that three drugs acted as plasticizers in the polymeric system, where ibuprofen contributed the most plasticizing effect (Figure 7). It is expected that drug molecules penetrate in the spaces between the polymer chains, which increases the free volume and decrease the *T_g_* of the polymeric system.

Recently, it has become more common to report the measured *T_g_* of particles used for drug delivery applications. In this review, the *T_g_* of PLGA nanoparticles from recent literature loaded with a drug are listed, along with the size, preparation, measurement, and heating rate, as shown in Table 1.

### 2.4. Water Content

**Figure 8:**
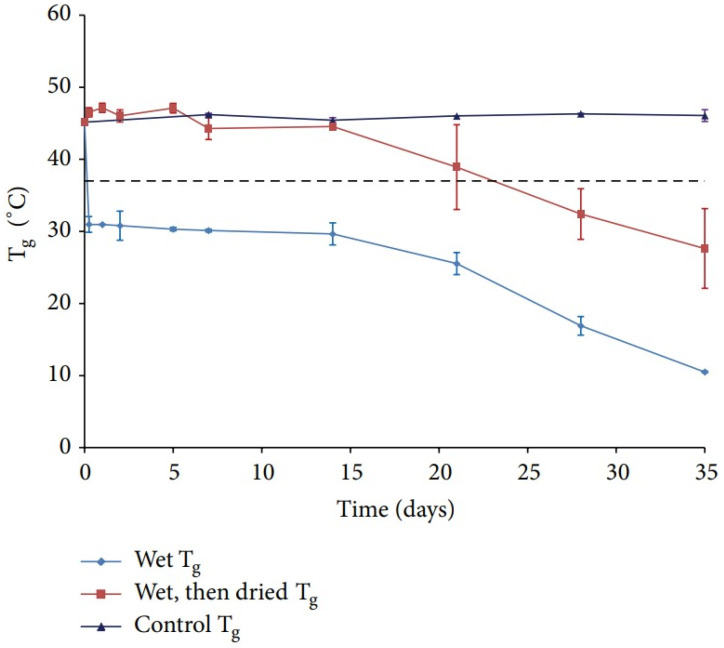
*T_g_* of PLGA under different post-treatment [93]. Reproduced from D’Souza, S. et al., Advances in Biomaterials 2014, 2014, under Creative Commons Attribution 4.0 International License (http://creativecommons.org/licenses/by/4.0/ (accessed on 13 February 2022)).

Water is well established as a plasticizer in polymeric systems [91] and it lowers the *T_g_* of many polymers. Passerini et al. reported that undried PLGA particles contain approximate 4.47% of moisture content and have a *T_g_* of 27.7 °C, which is about 15 °C lower than that of bulk PLGA polymer Notably, the dried particles which undergo 3 days’ lyophilization still contain 3.5% of residual moisture, and the *T_g_* is 33.1 °C [92]. Susan et al. further investigated the influence of water uptake on the *T_g_* of PLGA polymers. After incubation in 0.5% PVA solution, the dried PLGA polymer had a *T_g_* approximately 15 °C higher than the wet polymer, and *T_g_* recovery was achieved upon removing the moisture content (Figure 8). After 14 days’ incubation, lower PLGA chains were found, indicating that degradation of PLGA occurred [93].

### 2.5. Residual Surfactant

Amphiphilic compounds are widely utilized in PLGA particle manufacturing processes to generate monodisperse particles, reduce surface tension of the particles, and avoid aggregation among the particles [94]. The interaction between PLGA chains and other substances such as encapsulated drugs, trapped stabilizer, and residual solvent, will eventually influence the mobility of the PLGA matrix [95]. Sahoo et al. reported that it was quite challenging to remove the remaining poly (vinyl alcohol) (PVA) from PLGA particles before freeze-drying. A logical explanation would be that PVA filled in the inner pockets and coated the surface of PLGA particles [96]. The remining surfactant had an effect on the particle parameters of PLGA particles, including particle size, zeta potential, size distribution, surface hydrophobicity, and protein loading, and also had a modest effect on the encapsulated protein’s in vitro release. According to their report, the weight percentage of residual PVA could be up to 5% of PLGA particles prepared by emulsion-solvent evaporation technique [96]. Spek et al. illustrated the residual PVA present in PLGA particles by ^1^H nuclear magnetic resonance spectroscopy (NMR), which turned out to be 9.9 wt.% from the bulk of the particles. For the PEG-PLGA particles, PVA content could be as high as 35 wt.% on the particles surface based on the X-ray photoelectron spectroscopy (XPS) results [97]. In order to demonstrate the surfactant effect on the *T_g_* of polymeric particles, Feng et al. prepared PS nanoparticle with Brij 98 (nonionic surfactant) and sodium dodecyl benzene sulfonate (anionic surfactant) as well as surfactant-free particles. A substantial reduction in *T_g_* with decreasing size of surfactant-free particles was observed which corresponded to previous studies. On the other hand, the PS nanoparticles prepared with nonionic surfactant showed a weak correlation between size and *T_g_* reduction while anionic PS latex nanoparticles showed no correlation. The authors suggested that the incorporation of surfactant into the mobile layer influenced the free volume, thus affecting the *T_g_* of the particles [71].

## 3. Influence of *T_g_* on Drug Delivery

### 3.1. Particle Mobility

**Figure 9:**
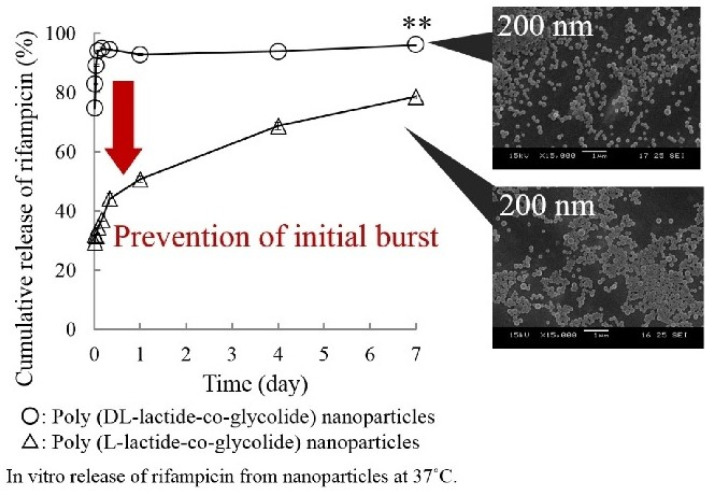
Effect of *T_g_* on drug release profiles [35]. Reprinted from Colloids and Surfaces A: Physicochemical and Engineering Aspects, 520, Takeuchi, I.; Tomoda, K.; Hamano, A.; Makino, K., Effects of physicochemical properties of poly(lactide-co-glycolide) on drug release behavior of hydrophobic drug-loaded nanoparticles, 771–778, Copyright (2017), with permission from Elsevier.

Takeuchi et al. examined the effects of the glass transition temperature on drug release behavior of drug loaded PLGA nanoparticles (Figure 9). 200 nm PLGA and PLLGA nanoparticles were prepared with a *T_g_* of 40.6 °C and 47.7 °C, respectively. The in vitro drug release study was carried out by dispersing the drug-loaded nanoparticles into PBS at 37 °C. More than 90% of the drug was released from the PLGA nanoparticles in the first two hours whereas only around 65% of the drug was released from PLLGA. The *T_g_* of PLLGA was 7 °C higher than PLGA and the crystallinity of these two samples was similar, which demonstrated that the *T_g_* strongly influenced the initial burst release [35].

**Figure 10:**
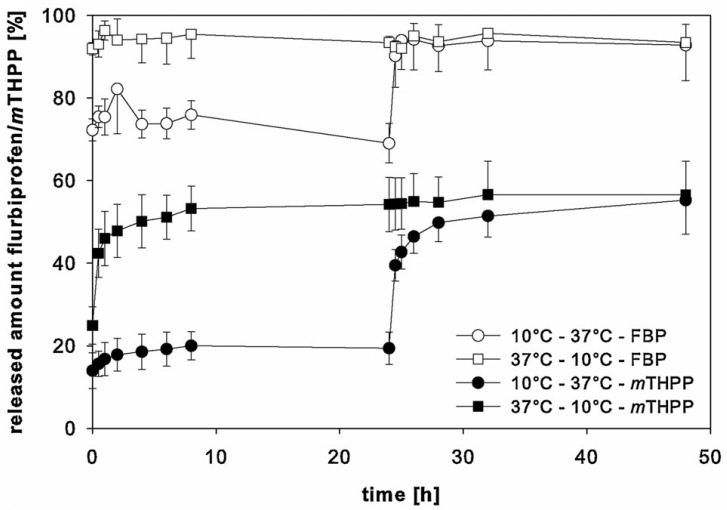
Release behaviors of drug loaded PLGA nanoparticles at different release temperatures [34]. Reprinted from International Journal of Pharmaceutics, 517, Lappe, S.; Mulac, D.; Langer, K., Polymeric nanoparticles—Influence of the glass transition temperature on drug release, 338–347, Copyright (2017), with permission from Elsevier.

Lappe et al. studied the correlation between *T_g_* and the release profile kinetics by comparing the release of two model drugs from PLGA nanoparticles at different temperatures (Figure 10). At the initial incubation temperature of 37 °C, the FBP-NPs reached around 93% drug release within a short time. Even after shifting the release medium temperature to 10 °C, the released amount of drug was constant at 19%. When the starting temperature was 10°C, only 70% of the drug was released after the first 24 h of incubation time, and an addition of 23% drug release was observed upon the changing the temperature to 37 °C. mTHPP-NPs had the same release behavior while the total amount of released drug was lower than FBP particles, which was mainly caused by the drug type. This study demonstrated that when the release medium temperature is lower than the *T_g_* of the nanoparticles, only the drug absorbed on the particle surface led to burst release, while at a higher temperature, the entrapped drug would also contribute to the burst release [34].

### 3.2. Physical Ageing of Particles

**Figure 11:**
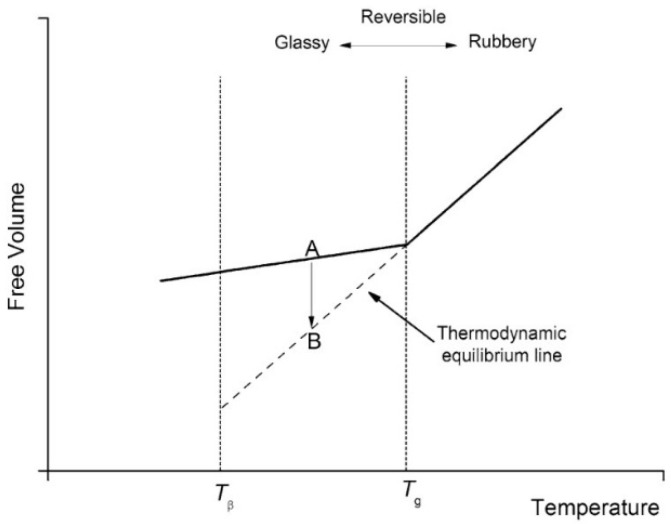
Explanation of structure relaxation in terms of free volume [104]. Reproduced from Motta Dias, M.H. et al., Mechanics of Time-Dependent Materials, 20, 2016, under Creative Commons Attribution 4.0 International License (http://creativecommons.org/licenses/by/4.0/ (accessed on 13 February 2022)).

**Figure 12:**
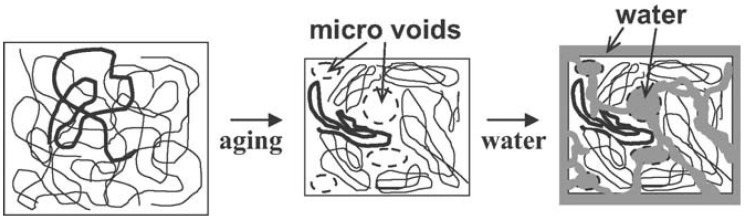
Appearance of micro spaces during polymer ageing process [102]. Reproduced with permission from Yoshioka, T. et al. Macromolecular Materials and Engineering, published by John Wiley and Sons, Copyright 2011.

Polymers are naturally non-equilibrium substances when they are in glassy states. During the process of cooling or solidification, polymer chains will reach a threshold where the thermal energy is inadequate for the polymer chains to rearrange on the given time scales [98]. As a result, the system loses its equilibrium and becomes arrested. The temperature where the glassy state develops is a cooling rate dependent parameter. Theoretically, the equilibrium state of a polymer can be achieved with an infinitely low cooling rate. Polymer in the glassy state, a non-equilibrium state, experiences a gradual relaxing process in order to achieve an equilibrium state, which is referred to as physical ageing or structural relaxation [99]. According to Figure 11, free volume decreases along with structural relaxation of the polymer (path A to B). As shown in Figure 12, in response to this phenomenon, the overall polymer matrix experiences shrinkage and micro spaces are created by rearranging of the local chains, which results in wide distribution of local density and helps the diffusion of water into the polymer matrix. Therefore, ageing time is very important to determine the penetration of water into polymeric particles. The time ($t_{\infty }$) needed to achieve thermodynamic equilibrium can be described in following equation [100]:

(3) t∞ ~ 100×10Tg−T3=100× e1.77(Tg−T)

According to the equation, ageing time is associated with the difference between $T_{g}$ of a given polymer and ageing temperature (T).

The extraction of solvent and solidification of PLGA chains during particle preparation is comparable to the process of quenching PLGA polymers [101,102]. Faster extraction of solvent leads to quicker rearrangement of PLGA chains; thus, particles will have more internal energy compared with those with slower extraction processes. The extra internal energy of PLGA particles is the driving force to relax the system toward thermodynamic equilibrium [103]. As mentioned before, when PLGA particles are placed in human body fluids, structural relaxation can be completed within a short time when the *T_g_* is close to 37 °C, which indicates that the micro spaces are created immediately after the administration of drug loaded PLGA particles. The improved water penetration will carry more drug molecules and enhance the diffusion, which leads to the initial burst release. Therefore, Kinam et al. stated that glass transition temperature and time it takes to complete structural relaxation significantly influence drug release behavior at the early stage.

### 3.3. Surface Reconfiguration

Hydration is the first and most important phase in the drug release process since water is required for drug disintegration and diffusion via the drug delivery system, whether in the form of biologic fluid or in vitro release medium [105]. Studies have shown that water penetration into PLGA particles can be completed within seconds through the porous structure [106]. The appearance of micro spaces during physical ageing of PLGA particles improves water absorption, resulting in an initial burst release of the drug. Water, on the other hand, acts as a plasticizer which decreases the glass transition temperature of PLGA particles, thus PLGA particles would be softer than the dry polymer state [92]. In addition, upon placement in release medium, a mobile layer forms on the particle surface, further softening the structure. All these plasticizing effects lead to surface reconfiguration, which closes the surface channels and inhibits the diffusion of drug and water penetration. This could explain the observed relative slow release rate following the initial burst [107].

## 4. Conclusions

**Figure 13:**
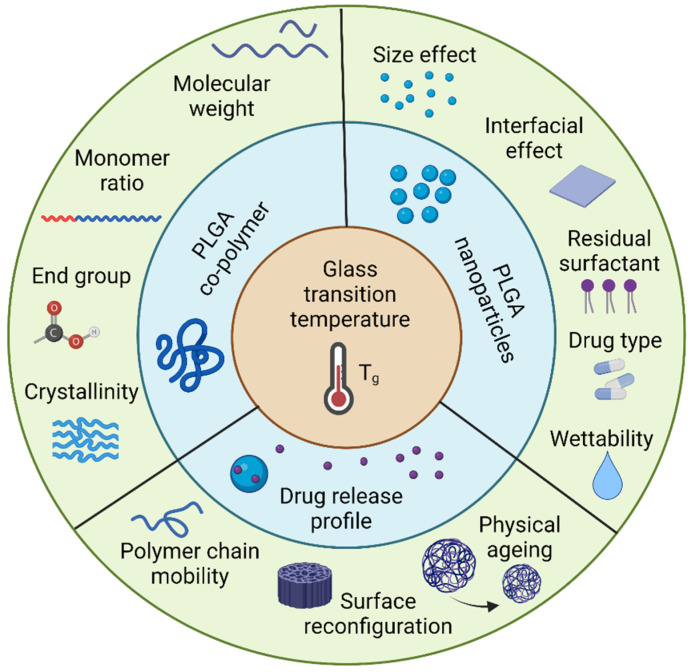
*T_g_* is an inherent property of PLGA nanoparticles that can predict the drug release profiles. Created with Biorender.com.

PLGA-based nanoparticles have received a great deal of interest as drug delivery vehicles for a variety of therapeutic purposes. It has been shown that the *T_g_* of polymeric nanoparticles has an impact on the drug release behavior, despite the fact that this physicochemical feature is often absent from many pharmaceutical research investigations. This review provides a comprehensive summary of variables affecting the *T_g_* of the PLGA copolymer, including molecular weight and monomer ratio. Additionally, research with PS particles was highlighted to demonstrate the size effect on the *T_g_* of polymeric particles and how that could affect PLGA particles. Drug type, moisture content, and residual surfactant are considerable parameters in altering *T_g_* during drug release processes. Finally, the connection of drug release and glass transition temperature are illustrated by three different aspects, which are the mobility of PLGA particles, ageing time on structural relaxation, and surface reconfiguration. The investigation into the effect of *T_g_* on drug release reveals that the *T_g_* of PLGA particles may account for the majority of the drug release profiles observed. In summary, the glass transition temperature, as an excellent indicator of drug release profiles, could be utilized in manufacturing PLGA particles for designed controlled drug release (Figure 13).

Looking forward in the field, the *T_g_* of particles for drug delivery should be reported in literature as it is critical to the behavior of the particles. Additionally, the factors affecting the *T_g_* should also be routinely reported to bring reproducibility to the particle synthesis process and consistent behavior in drug delivery applications.

## Figures and Tables

**Table 1:** Glass transition temperature of drug loaded PLGA nanoparticles.

PLGA LA:GA Mol wt. (g/mol)	Diameter (nm)	Model Drug	Preparation	*T_g_* (°C)	Measurement Heating Rate	Ref.
50:50 7000–17,000	Around 200	None	STM ^1^	39.35	DSC 10 °C/min	[85]
	Around 180	Atorvastatin	STM	42.49		
	Around 170	None	SUM ^2^	30.24		
	Around 190	Atorvastatin	SUM	35.02		
50:50 54,000–69,000	Around 240	None	STM	47.66	DSC 10 °C/min	[85]
	Around 230	Atorvastatin	STM	47.62		
	Around 225	None	SUM	25.98		
	Around 180	Atorvastatin	SUM	28.00		
85:15 Unknown	391+/−160	Menthol	W/O/W	48.0	DSC 10 °C/min	[86]
75:25 14,000 ^3^	162+/−3	None	Emulsion-evaporation	32.7+/−0.2	DSC 5 °C/min	[53]
75:25 32,000 ^3^	155+/−5	None	Emulsion-evaporation	37.6+/−0.2	DSC 5 °C/min	[53]
75:25 32,000 ^4^	213+/−18	None	Emulsion-evaporation	37.2+/−0.4	DSC 5 °C/min	[53]
75:25 14,000 ^4^	238+/−18	None	Emulsion-evaporation	24.8+/−0.6	DSC 5 °C/min	[53]
50:50 38,000–54,000	Unknown	Enrofloxacin	Emulsification-diffusion	32.9+/−0.8	MDSC 5 °C/min	[87]
	Unknown	None		31.26		
62:38 18,400	282+/−43	Insulin	Emulsification-diffusion	43.14	DSC 10 °C/min	[88]
50:50 Unknown	211.9+/−2	Abiraterone acetate	Modified single emulsion	45.64	DSC 5 °C/min	[89]
	170.9+/−2.1	Docetaxel		45.93		
	256.3+/−9.4	Abiraterone acetate/Docetaxel		46.61		
50:50 Unknown	179+/−13	Rutin	Single solvent evaporation	46.19	DSC 5 °C/min	[33]
	123+/−4	Rutin	Microfluidics	44.03		
75:25 Unknown	Unknown	Simvastatin	Emulsion solvent evaporation	51.5	DSC 10 °C/min	[90]
Unknown	226.8+/−6.8	Flurbiprofen	Emulsion diffusion	28.8+/−0.6	DSC 20 °C/min	[34]
	224.2+/−5.3	Flurbiprofen		26.9+/−0.5		
	222.8+/−4.8	Flurbiprofen		25.3+/−1.1		
	216.0+/−3.8	Flurbiprofen		22.4+/−1.5		
	223.3+/−11.7	Flurbiprofen		19.9+/−1.6		
	237.4+/−9.1	mTHPP		32.4+/−1.1		

^1^ Standard method: modified emulsion diffusion evaporation method. ^2^ Sustainable method: modified solvent displacement method. ^3^ PLGA polymer has acid terminal functional groups. ^4^ PLGA polymer has ester terminal functional groups.
